# Socioeconomic inequalities in maternal health service utilisation: a case of antenatal care in Nigeria using a decomposition approach

**DOI:** 10.1186/s12889-019-7840-8

**Published:** 2019-11-08

**Authors:** Chijioke O. Nwosu, John E. Ataguba

**Affiliations:** 1 Economic Performance and Development Unit, Human Sciences Research Council, Cape Town, South Africa; 2 Health Economics Unit, School of Public Health and Family Medicine, University of Cape Town, Cape Town, South Africa

**Keywords:** Socioeconomic inequality, Antenatal care, Decomposition, Nigeria

## Abstract

**Background:**

Antenatal care (ANC) services are critical for maternal health but Nigeria performs poorly in ANC utilisation compared to other countries in sub-Saharan Africa. This study aimed to assess socioeconomic inequalities in ANC utilisation and the determinants of these inequalities in Nigeria.

**Methods:**

The 2013 Nigeria Demographic and Health Survey data with 18,559 women was used for analysis. The paper used concentration curves and indices for different measures of ANC utilisation (no ANC visit, 1–3 ANC visits, at least four ANC visits, and the number of ANC visits). A positive (or negative) concentration index means that the measure of ANC utilisation was concentrated on the richer (poorer) population compared to their poorer (richer) counterparts. The concentration indices were also decomposed using standard methodologies to examine the significant determinants of the socioeconomic inequalities in no ANC visit, at least four ANC visits, and the number of ANC visits.

**Results:**

No ANC visit was disproportionately concentrated among the poor (concentration index (CI) = − 0.573), whereas at least four ANC visits (CI = 0.582) and a higher number of ANC visits (CI = 0.357) were disproportionately concentrated among the rich. While these results were consistent across all the geopolitical zones and rural and urban areas, the inequalities were more prevalent in the northern zones (which also have the highest incidence of poverty in the country) and the rural areas. The significant contributors to inequalities in ANC utilisation were the zone of residence, wealth, women’s education (especially secondary) and employment, urban-rural residence, ethnicity, spousal education, and problems with obtaining permission to seek health care and distance to the clinic.

**Conclusions:**

Addressing wealth inequalities, enhancing literacy, employment and mitigating spatial impediments to health care use will reduce socioeconomic inequalities in ANC utilisation in Nigeria. These factors are the social determinants of health inequalities. Thus, a social determinants of health approach is needed to address socioeconomic inequalities in ANC coverage in Nigeria.

## Introduction

Antenatal care (ANC) services are critical for maternal health, even in developing countries [1–3]. In 2016, the World Health Organization (WHO) introduced a new recommendation that pregnant women with uncomplicated pregnancies should attain at least eight ANC contacts [4]. Previously, a minimum of four ANC visits (ANC4+) was recommended [4, 5]. Although the WHO recommends a new minimum, many official statistics and international documents still provide ANC indicators using ANC4+ [6]. In some contexts, especially in sub-Saharan Africa, the indicator of attaining at least one ANC visit remains relevant [7]. Although these indicators do not address the issue of quality of the ANC services [8–11], they still indicate service coverage.

**Fig. 1:**
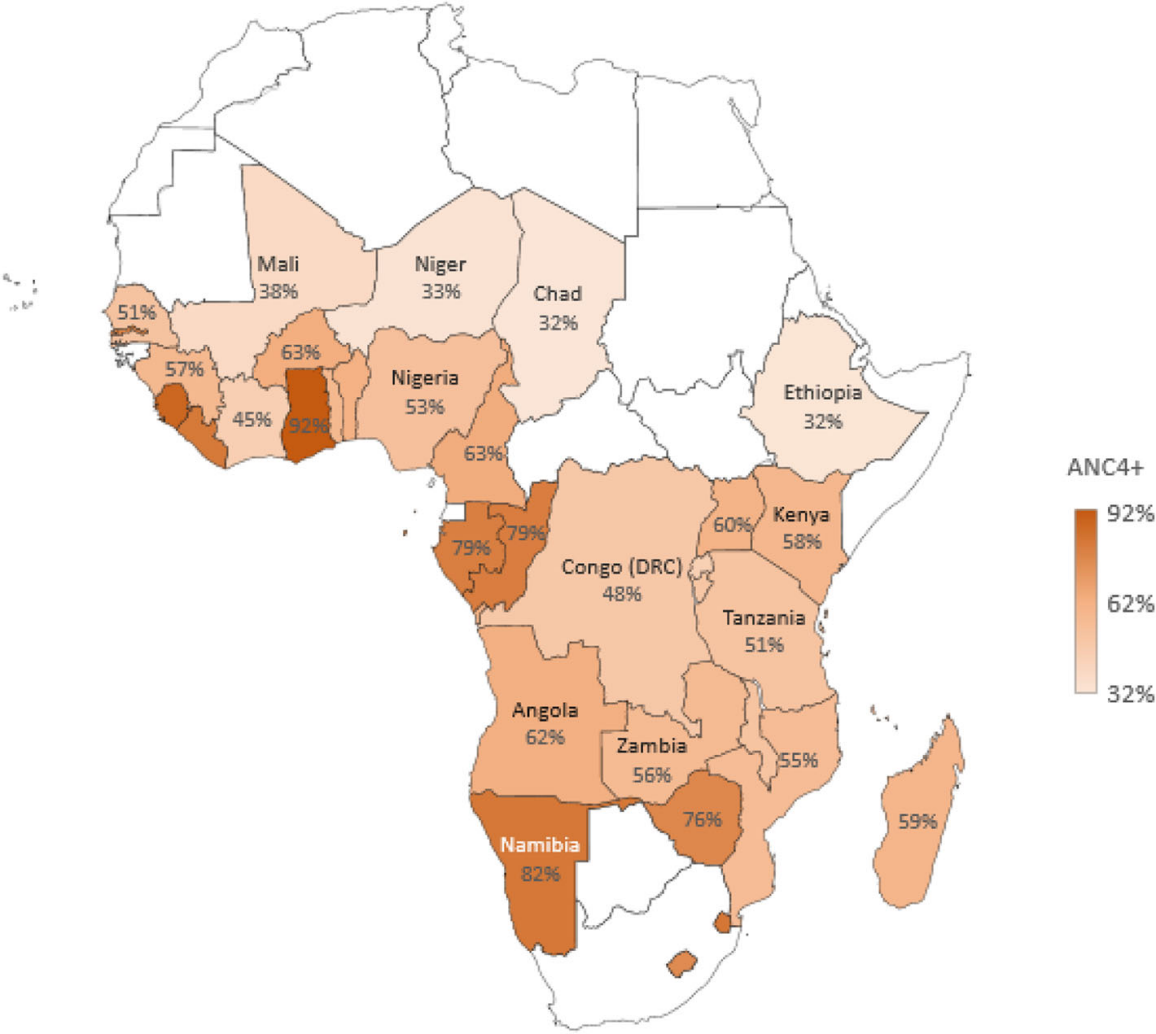
Proportion of women in sub-Saharan Africa attending at least four ANC visits. Source: Adapted from Ataguba JE [6]. Plotted using original data points with permission from Ataguba J.E., PLOS One; published by Public Library of Science; CC BY 4.0; 10.1371/journal.pone.0204822. *Note*: White spaces mean that data are not available or reliable

Nigeria, sub-Saharan Africa’s most populous country, still has a relatively low proportion (53%) of pregnant women who attend at least four ANC visits (Fig. 1) compared to other countries in the region. Frequently cited reasons for non-utilisation of ANC services in Nigeria include affordability, availability, and accessibility [12]. Also, the country sits with a very high maternal mortality ratio (814 per 100,000 live births in 2015 with uncertainty interval ranging from 596 to 1180 maternal deaths per 100,000 live births) that is higher than the average for sub-Saharan Africa (765 per 100,000 live births) [13]. Although regional differences exist in these statistics, to the disadvantage of the more impoverished regions in Nigeria [14, 15], the country alone accounts for about one-seventh of global maternal mortality [16]. A recent Lancet study ranked Nigeria 142 out of 195 countries in terms of health access and quality of health care, ranking worse than some conflict and post-conflict states [17]. This means that the country has significant challenges to address to substantially improve access to health services and increase the uptake of ANC services.

Some studies show that ANC service utilisation can be associated with residential location [2], wealth [14, 18], maternal educational attainment [19], the woman’s age [20], religion [21], spousal education and women’s autonomy [19, 22, 23], among others. However, there is a dearth of studies that assess socioeconomic inequality in ANC coverage which look at the intensity of service coverage and no service coverage. An initial attempt at such categorisation in Nigeria was made for immunisation coverage [15]. Thus, this paper assesses socioeconomic inequalities in ANC visits in Nigeria and within the country’s six geopolitical zones,^1^ and rural and urban locations given the well-known regional differences in maternal health service utilisation [2, 14]. Further, to deepen our understanding and to highlight areas for policy action, the paper assesses factors that significantly explain socioeconomic inequalities in ANC visits in the country using a decomposition approach.

## Methods

### Data and key variables

Data came from the nationally representative 2013 Nigeria Demographic and Health Survey (NDHS). The NDHS used a stratified three-stage cluster design consisting of 904 primary sampling units (PSUs) (i.e. 372 urban and 532 rural PSUs) and was designed to provide indicators at the national, zonal, and state levels. The NDHS selected 40,680 households, with a minimum of 943 completed interviews per state. A detailed explanation of the survey is available elsewhere [25]. This study is based on the Individual Recode file, which focuses on women of reproductive age (15–49 years). Due to missing observations in some of the variables used in the analysis, the final sample used in this paper consisted of 18,559 women. The proposed analyses to address this paper’s objectives are different from those already contained in the NDHS report.

The key variables analysed in this study are three measures of ANC utilisation. Each woman who had been pregnant within the last five years was asked about her ANC service utilisation. The question elicited the number of ANC visits made by the woman. Based on this question, we derived three measures of ANC utilisation— (i) *no ANC visit*, a dummy variable for no ANC visit, (ii) *ANC4+*, a dummy variable for a woman that made at least 4 ANC visits, (iii) *ANC visit intensity*, a count variable for the number of ANC visits. A fourth variable, *1–3 ANC visits*, a dummy variable for a woman with between one and three ANC visits was also included for assessing socioeconomic inequality only and was not used in the decomposition analysis.^2^ Table 1 contains the full list of the variables used in the paper and their description.

**Table 1:** Description of variables and descriptive statistics

Variable	Variable description	Mean/ Proportion
*Antenatal visits during pregnancy*		
No ANC visit	= 1 if the woman did not visit any antenatal clinic; 0 otherwise	35.4%
1–3 ANC visits	= 1 if the woman made either one or two or three ANC visits; 0 otherwise	12.4%
ANC4+	= 1 if the woman made at least four ANC visits; 0 otherwise	52.2%
ANC visit intensity	Number of ANC visits	5.1
Distance to a clinic is a big problem	= 1 if the woman feels that the distance to the nearest clinic is a big problem; 0 if she feels it is not a big problem	31.9%
*Woman’s highest educational attainment*		
No education	= 1 if the woman did not attend school; 0 otherwise	49.4%
Some primary education	= 1 if the woman has some primary education; 0 otherwise	19.1%
Some secondary education	= 1 if the woman has some secondary education; 0 otherwise	25.4%
Higher education	= 1 if the woman has some higher education; 0 otherwise	6.1%
Woman’s age	Woman’s age in years	29.6
Married/cohabiting	= 1 if the woman is married/living with a partner; 0 otherwise	97.0%
Household head	= 1 if the woman is a household head; 0 otherwise	6.5%
Woman is employed	= 1 if the woman is working; 0 otherwise	69.2%
Urban residence	= 1 if the woman lives in an urban area; 0 if she lives in a rural area	35.0%
Problem obtaining permission to seek medical help	=1 if obtaining permission for the woman to seek own health care is a big problem; 0 if it is not a big problem	12.6%
Attitude of health workers	= 1 if health worker attitude is a big problem; 0 otherwise	16.9%
*Household wealth categories*		
1. poorest	= 1 if the woman belongs to the poorest quintile; 0 otherwise	23.7%
2. poorer	= 1 if the woman belongs to the poorer quintile; 0 otherwise	22.8%
3. middle	= 1 if the woman belongs to the middle quintile; 0 otherwise	18.8%
4. richer	= 1 if the woman belongs to the richer quintile; 0 otherwise	17.5%
5. richest	= 1 if the woman belongs to the richest quintile; 0 otherwise	17.2%
*Religion*		
Islam	= 1 if the woman is a Muslim; 0 otherwise	62.9%
Catholic	= 1 if the woman is a Catholic; 0 otherwise	8.4%
Other Christian	= 1 if the woman is a non-Catholic Christian; 0 otherwise	27.7%
Traditional/Other	= 1 if the woman practices traditional/other religion; 0 otherwise	1.1%
Ethnicity	= 1 if the woman is Igbo/Yoruba; 0 otherwise	22.6%
*Geopolitical zone*		
North-Central	= 1 if the woman comes from the North-Central; 0 otherwise	14.2%
North-East	= 1 if the woman comes from the North-East; 0 otherwise	17.1%
North-West	= 1 if the woman comes from the North-West; 0 otherwise	37.6%
South-East	= 1 if the woman comes from the South-East; 0 otherwise	8.0%
South-South	= 1 if the woman comes from the South-South; 0 otherwise	8.5%
South-West	= 1 if the woman comes from the South-West; 0 otherwise	14.6%
Spouse employment	= 1 if the spouse is working; 0 otherwise	99.2%
*Spouse’s highest educational attainment*		
No education	= 1 if the spouse has no education; 0 otherwise	39.9%
Primary education	= 1 if the spouse has primary education; 0 otherwise	18.4%
Secondary education	= 1 if the spouse has secondary education; 0 otherwise	29.1%
Higher education	= 1 if the spouse has some higher education; 0 otherwise	12.6%
Sample size		18,559

*Note*: Estimates are weighted by the women’s sampling weight

A woman’s socioeconomic status was assessed using the traditional DHS wealth index [25] based on the household asset data [26]. The principal components analysis approach was used, and this accounted for urban-rural differences in the wealth scores. Where necessary, quintiles of socioeconomic status were generated from the wealth index. The wealth index was used as a measure of socioeconomic status because the DHS does not contain suitable data on household income or expenditure.

### Analytical methods

#### Concentration curves

Socioeconomic inequality was depicted using concentration curves. A concentration curve depicts the cumulative shares of each measure of ANC utilisation against the cumulative population shares, ranked by socioeconomic status. A 45-degree diagonal line depicts a line of equality. A pro-poor concentration curve is one that lies above the line of equality, as the measure of ANC utilisation is disproportionately concentrated on the poor. A pro-rich concentration curve is an opposite of a pro-poor curve [27]. A proportional concentration curve is one that theoretically coincides with the line of equality.

#### Concentration indices

Socioeconomic inequality was also assessed using concentration indices. The concentration index (*I*_*H*_) was computed as follows [28]:

1 $$ {I}_H=1-\left({\hat{\xi}}_H/{\hat{\mu}}_H\right) $$

where ${\hat{\mu}}_H$ is the weighted average for each measure of ANC utilisation (binary or count), ${\hat{\xi}}_H={\sum}_{i=1}^n\left(\left({\left({V}_i\right)}^2-{\left({V}_{i+1}\right)}^2\right)/{\left({V}_1\right)}^2\right){h}_i$, where ${V}_i={\sum}_{h=i}^n{w}_h$. The vector **w** = [*w*_1_, *w*_2_, …, *w*_*n*_] is the sampling weights and the socioeconomic status (i.e. the ranking variable), **X**, is such that *x*_1_ ≥ ⋯ ≥ *x*_*n* − 1_ ≥ *x*_*n*_.

The values of the concentration index range between −1 and + 1 [27]. A negative index corresponds to a pro-poor concentration curve; a positive index is equivalent to a pro-rich concentration curve whereas *I*_*H*_ = 0 implies perfect equality and is akin to the concentration curve coinciding with the line of equality [27]. For categorical variables, the concentration index is not bounded between − 1 and + 1, thus requiring some normalisation [29]. This paper adopts the Erreygers’ normalised concentration index (*E*_*I*_) [30, 31] that can be obtained as [32]:

2 $$ {E}_I=4\left(\frac{\mu_H}{b-a}\right){I}_H $$

where *b* and *a* indicate the upper and lower bounds of the ordinal measures of ANC utilisation, respectively. The original concentration index (*I*_*H*_) is used to assess socioeconomic inequality in *ANC visit intensity* while the Erreygers normalised index is used for the other measures of ANC utilisation.

#### Inequality decomposition

Beyond using the concentration indices and curves, policymakers are interested in the factors that drive socioeconomic inequalities. The approach developed in Wagstaff A, van Doorslaer E and Watanabe N [33] was used to provide this evidence.

We denote the relationship between any of the measures of ANC utilisation (*H*) and relevant socioeconomic and demographic factors (*z*) as follows:

3 $$ {H}_i=\alpha +{\sum}_k{\beta}_k{z}_{ki}+{\varepsilon}_i $$

where *α* and *β* are parameters, and *ε* is the error term.

The concentration index in eq. (1) can be re-written as:

4 $$ {I}_H={\sum}_{k=1}^K\left(\frac{\beta_k{\overline{z}}_k}{\mu_H}\right){I}_k+\left(\frac{G{I}_{\varepsilon }}{\mu_H}\right) $$

where *I*_*H*_ and *μ*_*H*_ remain as earlier defined. $\left(\frac{\beta_k{\overline{z}}_k}{\mu_H}\right)$ represents the elasticity of ANC utilisation to marginal changes in the *k*-th explanatory variable, while *I*_*k*_ refers to the concentration index of the *k*-th explanatory variable. *GI*_*ε*_ denotes the generalized concentration index of the error term. $\left(\frac{\beta_k{\overline{z}}_k}{\mu_H}\right){I}_k$ represents the contribution of the *k*-th explanatory variable to the socioeconomic inequality in the measure of ANC utilisation (in this case, *no ANC visit*, *ANC4+*, or *ANC visit intensity*). The term, $\left(\frac{G{I}_{\varepsilon }}{\mu_H}\right)$, captures the unexplained/residual component.

Apart from the concentration index where analytical standard errors were computed directly, the standard errors for the various components of the decomposition in eq. 4 were computed using the bootstrap method [34, 35]. This is because analytical standard errors do not exist for these composite components. Bootstrapped standard errors were computed with 1000 replications [36], taking the NDHS’s full sampling structure into account. Stata® software [36] was used for estimations, and in some cases via the DASP routine [28].

## Results

The average age of the women was about 30 years, whereas most of the women (97%) were either married or living with a partner (Table 1). Most of the women (65%) resided in rural areas, with North-West and North-Central accounting for more than half of the women population. Over 35% of the population had no ANC visit during their last pregnancy in the past five years. However, over 52% achieved at least four visits. While close to half of the women population did not have any formal education, and less than 7% had tertiary education, education attainment was better for their spouses. Also, only 6.5% of the women were household heads with double of that proportion of women (13%) expressing a challenge with obtaining permission to seek health care. Employment status was generally better among men (99.2%) compared to the women population (69.2%).

**Fig. 2:**
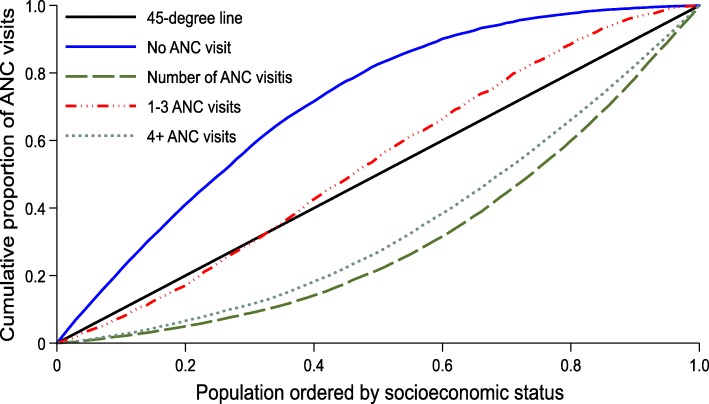
Concentration curves for ANC visits, Nigeria, 2013. *Source*: Authors’ computation

Figure 2 indicates that women with no ANC visit were disproportionately concentrated among the poor since the concentration curve lies above the line of equality. On the other hand, there was a pro-rich distribution of women with at least four ANC visits as they are concentrated among the rich. On average, richer women record more ANC visits than poorer women as the concentration curve of ANC visit intensity lies below the line of equality. The distribution of women with between 1 and 3 ANC visits was also pro-poor for most parts. Taken together, the results in Fig. 2 indicate that in Nigeria, poorer women have fewer or no ANC visits compared to richer women.

The results in Table 2 confirm those in Fig. 2 as there was a statistically significant pro-poor distribution of women with no ANC visit (concentration index = − 0.573). Significant pro-rich distributions are observed for ANC4+ (concentration index = 0.582) and ANC visit intensity (concentration index = 0.357). The patterns observed in the concentration indices are consistent across different geographic locations shown in Table 2—No ANC visits are significantly pro-poor, whereas ANC4+ and ANC visit intensity are significantly pro-rich. The magnitude of pro-poorness or pro-richness varies by location, depending on the measure of ANC visits. Generally, the northern geopolitical zones and rural areas had the highest estimate of socioeconomic inequality, whether it is pro-poor or pro-rich.

**Table 2:** Concentration indices by geopolitical zone and urban-rural location, Nigeria, 2013

Variables	No ANC visit	ANC4+ visits	ANC visit intensity
*Geopolitical zone*			
North-Central	− 0.462*** (0.041)	0.456*** (0.083)	0.246*** (0.032)
North-East	− 0.518*** (0.054)	0.382*** (0.054)	0.235*** (0.030)
North-West	− 0.468*** (0.045)	0.396*** (0.030)	0.303*** (0.022)
South-East	−0.087*** (0.020)	0.192*** (0.030)	0.090*** (0.016)
South-South	−0.224*** (0.038)	0.339*** (0.042)	0.171*** (0.020)
South-West	−0.163*** (0.016)	0.226*** (0.075)	0.076*** (0.024)
*Location*			
Urban	−0.224*** (0.015)	0.324*** (0.036)	0.156*** (0.015)
Rural	−0.514*** (0.023)	0.451*** (0.027)	0.360*** (0.017)
Population	−0.573*** (0.016)	0.582*** (0.024)	0.357*** (0.012)

*Note*: Estimation sample was 18,559; estimates are weighted by the women’s sampling weight; *E*_*I*_ computed for all dummy outcomes; standard errors in parenthesis; *, **, and *** indicate statistical significance at the 10, 5, and 1% respectively

The decomposition results in Table 3 show that the significant contributors to socioeconomic inequality in no ANC visit included the geopolitical zone of residence, wealth, woman’s education, urban residence, ethnicity, the problem with the distance to the clinic, the problem with permission to seek medical help, and spousal secondary and higher education. Apart from a few, a similar set of variables contribute significantly to socioeconomic inequalities in ANC4+ and ANC visit intensity. Because the overall concentration index for no ANC visit was negative, any significant negative contributor in Table 3 means that socioeconomic inequality in no ANC visit would have been less pro-poor if: (i) the contributing variables (e.g. employment or urban location) were to be evenly distributed among the rich and poor (i.e. a case where the concentration index = 0) and/or (ii) the elasticity were to be zero. For the positive contributing factors, they would otherwise contribute towards a more pro-poor socioeconomic distribution of no ANC visit if the concentration index of the contributing variable was zero and/or the elasticity was zero.

**Table 3:** Factors that explain socioeconomic inequalities in ANC service utilisation, Nigeria, 2013

	Concentration index	Elasticity			Contribution		
		No ANC visit	ANC4+	ANC use intensity	No ANC visit	ANC4+	ANC visit intensity
Age	0.012*** (0.001)	−0.069* (0.038)	0.111*** (0.028)	0.105*** (0.030)	−0.001* (0.000)	0.001*** (0.000)	0.001*** (0.000)
Primary education	0.073*** (0.007)	−0.067*** (0.006)	0.032*** (0.005)	0.026*** (0.004)	−0.005*** (0.001)	0.002*** (0.000)	0.002*** (0.000)
Secondary education	0.464*** (0.008)	−0.105*** (0.009)	0.065*** (0.007)	0.067*** (0.008)	−0.049*** (0.004)	0.030*** (0.003)	0.031*** (0.004)
Higher education	0.189*** (0.006)	−0.025*** (0.003)	0.017*** (0.002)	0.021*** (0.003)	−0.005*** (0.001)	0.003*** (0.000)	0.004*** (0.001)
Married/cohabiting	−0.010*** (0.003)	− 0.030 (0.051)	− 0.001 (0.038)	0.054 (0.051)	< 0.001 (0.001)	< 0.001 (0.000)	− 0.001 (0.001)
Distance big problem	−0.366*** (0.008)	0.082*** (0.008)	−0.062*** (0.005)	− 0.059*** (0.005)	− 0.030*** (0.003)	0.023*** (0.002)	0.022*** (0.002)
Poorer	−0.272*** (0.007)	−0.082*** (0.007)	0.038*** (0.004)	0.027*** (0.003)	0.022*** (0.002)	−0.010*** (0.001)	−0.007*** (0.001)
Middle	0.088*** (0.006)	−0.118*** (0.007)	0.067*** (0.005)	0.050*** (0.004)	−0.010*** (0.001)	0.006*** (0.001)	0.004*** (0.000)
Richer	0.337*** (0.007)	−0.131*** (0.008)	0.081*** (0.005)	0.080*** (0.005)	−0.044*** (0.003)	0.027*** (0.002)	0.027*** (0.002)
Richest	0.570*** (0.009)	−0.117*** (0.008)	0.086*** (0.006)	0.111*** (0.007)	−0.067*** (0.005)	0.049*** (0.004)	0.063*** (0.004)
Employed	0.165*** (0.009)	−0.074*** (0.014)	0.042*** (0.010)	0.056*** (0.010)	−0.012*** (0.003)	0.007*** (0.002)	0.009*** (0.002)
Urban	0.689*** (0.007)	−0.070*** (0.008)	0.026*** (0.007)	−0.004 (0.008)	−0.048*** (0.006)	0.018*** (0.005)	−0.003 (0.006)
Household head	0.064*** (0.005)	−0.002 (0.002)	0.003** (0.002)	0.012*** (0.003)	<−0.001 (0.000)	< 0.001** (0.000)	0.001*** (0.000)
Permission big problem	−0.147*** (0.006)	0.043*** (0.004)	−0.020*** (0.003)	−0.011*** (0.002)	− 0.006*** (0.001)	0.003*** (0.000)	0.002*** (0.000)
Catholic	0.077*** (0.006)	0.012*** (0.003)	−0.006*** (0.002)	−0.022*** (0.003)	0.001*** (0.000)	<−0.001*** (0.000)	− 0.002*** (0.000)
Non-Catholic	0.395*** (0.008)	0.028*** (0.006)	−0.001 (0.005)	0.012 (0.008)	0.011*** (0.002)	<−0.001 (0.002)	0.005 (0.003)
Traditional/other	−0.011*** (0.002)	0.004*** (0.001)	−0.002*** (0.001)	−0.001 (0.001)	<− 0.001*** (0.000)	< 0.001** (0.000)	< 0.001 (0.000)
Health worker attitude	−0.116*** (0.007)	− 0.010** (0.005)	0.007** (0.003)	− 0.009*** (0.003)	0.001** (0.001)	− 0.001** (0.000)	0.001*** (0.000)
Spouse: primary	−0.002 (0.007)	−0.076*** (0.007)	0.044*** (0.004)	0.021*** (0.004)	< 0.001 (0.001)	<−0.001 (0.000)	<−0.001 (0.000)
Spouse: secondary	0.391*** (0.008)	−0.111*** (0.010)	0.075*** (0.007)	0.054*** (0.007)	−0.043*** (0.004)	0.029*** (0.003)	0.021*** (0.003)
Spouse: higher	0.286*** (0.007)	−0.060*** (0.005)	0.041*** (0.004)	0.027*** (0.004)	−0.017*** (0.002)	0.012*** (0.001)	0.008*** (0.001)
Spouse: employed	−0.010*** (0.002)	−0.137 (0.090)	− 0.037 (0.071)	−0.139 (0.107)	0.001 (0.001)	< 0.001 (0.001)	0.001 (0.001)
Ethnicity fixed effect	0.463*** (0.008)	−0.046*** (0.005)	0.072*** (0.004)	0.207*** (0.007)	−0.021*** (0.002)	0.033*** (0.002)	0.096*** (0.004)
Geopolitical zone fixed effect	0.412*** (0.009)	−0.182*** (0.016)	0.097*** (0.011)	0.151*** (0.009)	−0.075*** (0.007)	0.040*** (0.005)	0.062*** (0.004)
Residual					−0.176*** (0.012)	0.309*** (0.007)	0.010* (0.006)
Total					−0.573*** (0.016)	0.582*** (0.024)	0.357*** (0.012)

Notes: Estimation sample was 18,559; estimates are weighted by the women’s sampling weight; standard errors in parenthesis; bootstrapped standard errors with 1000 replications for elasticity and contributions;

*, **, and *** indicate statistical significance at the 10, 5, and 1%, respectively

Similarly, the overall positive concentration indices for ANC4+ and ANC visit intensity mean that any explanatory variable in Table 3 with a positive contribution (e.g. age and education) would have reduced the pro-rich distribution of ANC4+ or ANC visit intensity if the concentration index of the explanatory variable and/or elasticity in Table 3 was zero. Negative contributing variables would cause the opposite effect.

Marital status and spouse’s employment status had minimal contributions to the socioeconomic inequalities in ANC utilisation. Because some unexplained factors affect ANC utilisation, the residuals came up significant for no ANC visit and ANC4 + .

**Fig. 3:**
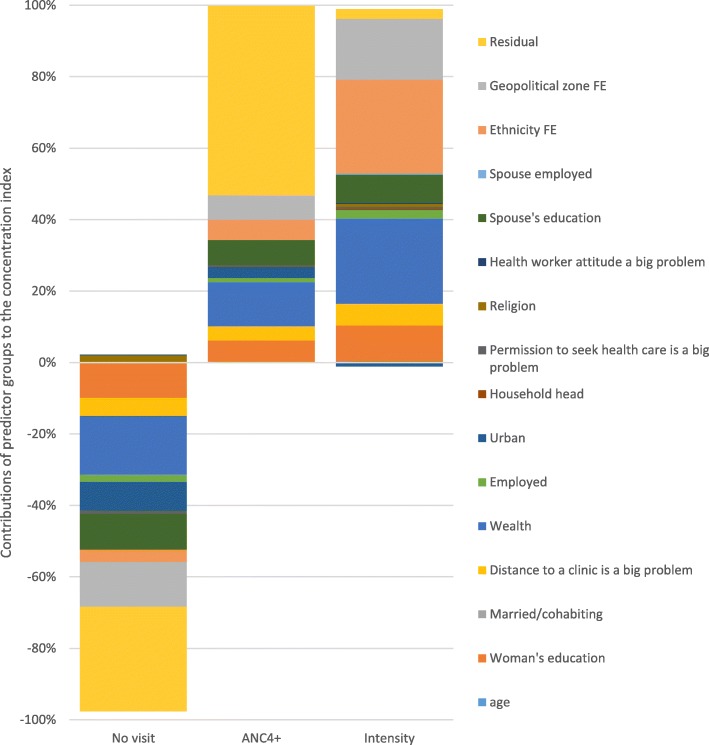
Percentage contributions of factors explaining socioeconomic inequalities in ANC visits, Nigeria, 2013. *Source*: Authors’ computation

Figure 3 depicts the contributions of various predictor groups to the socioeconomic inequalities in ANC visits (here, the contributions of the components of a group of variables, e.g. the wealth quintiles, are added up). It indicates that for no ANC visit and ANC4+ measures, the group of known determinants with the most significant contribution was household wealth. Wealth only came second for ANC visit intensity, with ethnicity being the most dominant determinant. We also found that the contribution of spousal education was similar to women’s education, especially for no ANC visit; indeed, the total contribution of spousal education exceeded that of women’s education for both no visit and ANC4+ visits.

## Discussion

This study has shown that significant socioeconomic inequalities exist in the use of ANC services in Nigeria, in favour of the rich. Non-utilisation of ANC services was more than proportionately prevalent among the poor, while richer women in Nigeria report having at least four ANC visits during pregnancies compared to their poorer counterparts. Also, the number of ANC visits recorded by women increased for richer women relative to poorer women. Socioeconomic inequalities in ANC visits across the six geopolitical zones were similar in pattern to those observed for the entire population. However, inequalities were more substantial in the northern geopolitical zones, with the highest poverty rates in the country [24], as well as in the rural areas. The study also identified salient factors that explained some of these socioeconomic inequalities including wealth, the geopolitical zone of residence, education status of women and their spouses, ethnicity, distance to a clinic, and urban/rural residence.

The proportion of women not receiving any ANC during pregnancy as estimated in this paper was substantial—over one-third—and this was prevalent among poorer women in Nigeria. A higher percentage (> 62%) was recorded in a selection of four rural states in northern India [19]. Pallikadavath S, Foss M and Stones RW [19], using women’s education status as a measure of socioeconomic status in a regression model, also showed that poorer women in rural India were more likely to have had no ANC visit during pregnancy. The socioeconomic gradients in ANC utilisation are also similar for the use of multiple maternal health services, including skilled birth attendants [2, 37].

The results of the decomposition analysis in this paper highlight some important things. A significant policy lever was education, especially women’s education. By eliminating inequality in women’s education, the concentration of women with no ANC visit will reduce by 10.3% among the poor. The effects are more substantial for achieving equality in female secondary and tertiary education in Nigeria. Currently, the female literacy rate is low in northern Nigeria, a region with higher levels of socioeconomic inequalities in ANC coverage. In the North-West geopolitical zone, for instance, female literacy is as low as 38% [38]. Similarly, addressing the inequality in female secondary school attainment will reduce the extent to which attaining at least four ANC visits disproportionately favour the rich by 5.2% as this will increase the proportion of poorer women attaining at least four ANC visits. This was also the case for the analysis using the number of ANC visits. These analyses highlight the critical importance of maternal education and its impact on maternal health service utilisation as reported elsewhere [19]. Relatedly, this paper found a significant relationship between husband’s education and ANC attendance and this has also been reported elsewhere [22, 39]. In the Philippines, for example, a husband’s education had a stronger association with ANC utilisation than the woman’s education [40].

Nigeria’s education system currently provides for free and compulsory education in public schools for the first nine years of schooling (i.e. up to the first three years of secondary school) [41]. However, literacy levels remain low in many parts of the country as this policy has not been effective in ensuring high literacy rates, especially among women in some of the northern geopolitical zones. Thus, there is the need to understand and address the barriers to the effective implementation of this policy and possibly extend it to the remaining years of secondary education, especially for females given the substantial contribution of secondary education to the reduction in inequalities in ANC utilisation in Nigeria.

The results in this paper show that achieving significant reductions in wealth inequality would reduce the socioeconomic inequalities in ANC utilisation significantly. A recent Oxfam study has shown that the combined wealth of the country’s five richest men (US$ 29 billion) could end extreme poverty in Nigeria, while the annual earning of the wealthiest Nigerian can lift two million Nigerians out of poverty for a year [42]. Moreover, data from the World Bank and the United Nations Development Programme indicate that the Gini index for Nigeria —a measure of inequality, with larger values denoting higher income inequality—rose from 0.401 in 2003 to 0.488 in 2013 [43, 44]. Based on the findings in this paper, rising levels of income and wealth inequality will substantially widen socioeconomic inequalities in ANC service utilisation to the disadvantage of the poor. As shown in this paper, eliminating the current levels of wealth inequality will reduce socioeconomic inequality in no ANC visit, ANC4+, and the number of ANC visits by 17.3, 12.4, and 24.4%, respectively.

Place of residence is also a salient determinant of the socioeconomic inequalities in ANC visits. Geopolitical fixed effects contributed 13.1, 6.9, and 17.4% to the inequalities in no ANC visit, ANC4+, and ANC visit intensity, respectively. As discussed earlier, these results imply that poor women lived in zones that were likely to predispose them to not utilising adequate ANC services. With higher poverty levels in northern Nigeria [24], and most of the women in the study coming from the northern zones, it is not surprising that geopolitical zone of residence had a significant effect on socioeconomic inequalities in ANC utilisation. A deeper understanding of the contextual factors which predispose the northern zones to low ANC utilisation may be required to address the impact of geographic location on socioeconomic inequality in ANC coverage. It is essential to understand how such contextual factors directly affect ANC utilisation and modify the determinants identified in this paper and those reported in previous studies [45].

This study has some strengths and limitations. A significant strength of the paper is the innovative approach used in categorising ANC service utilisation. Through this process, the paper can uncover differences between socioeconomic inequalities in no ANC visits, ANC4+ visits and ANC visit intensity. The decomposition of the factors that drive socioeconomic inequalities in the different measures of ANC utilisation provided a rich set of analysis for policy interventions to address socioeconomic inequalities in ANC coverage in Nigeria. However, a shortcoming of this study is the use of a linear model, as opposed to a non-linear model, for the decomposition analysis of no ANC visit and ANC4+. As noted elsewhere, this may have a very negligible impact on the results [46]. Another limitation was the magnitude and statistical significance of the residuals or unexplained factors, especially for ANC4+ visits and no ANC visit. Ideally, the residual should be close to zero in a model that controls for all factors that affect ANC utilisation [27]. However, in this case, the significant residuals mean that there are factors that remain unexplained and as explained elsewhere in northern Nigeria, many mothers were not able to articulate specific reasons for the lack of immunisation for their children [47]. While these factors may not be captured using any quantitative analysis, there is a need for a detailed qualitative assessment to uncover barriers to accessing ANC services that go beyond those considered in this paper. A critical factor could be the quality of ANC services [17, 48–50]. Also, future research could examine changes in socioeconomic inequalities in ANC utilisation and assess factors that are associated with these changes.

## Conclusion

Socioeconomic inequalities in the use of ANC services disproportionately favour the rich in Nigeria. Although socioeconomic inequalities are more substantial in the rural areas and the northern parts of Nigeria, richer women enjoy relatively more ANC services than their poorer counterparts irrespective of their geographic location. Significant factors such as household wealth, location (geopolitical zone and urban residence), employment and education significantly explained the socioeconomic inequalities. These factors are the social determinants of health inequalities, suggesting that a social determinants of health approach to reducing socioeconomic inequalities in ANC coverage is required in Nigeria. Also, it is crucial to understand the unexplained factors that have not been included in the analysis in this paper. Using deliberate attempts to redressing inequalities in maternal health will *leave no one behind* and contribute towards the attainment of universal health coverage as entrenched in the Sustainable Development Goals.

## Data Availability

The 2013 Nigeria Demographic and Health Survey dataset used in this article is available in the DHS repository, https://dhsprogram.com/data/dataset/Nigeria_Standard-DHS_2013.cfm?flag=0. Data are accessible after registration on the website.

## Abbreviations

ANC: Antenatal care
ANC4 +: At least four ANC visits
CI: Concentration index
NDHS: Nigeria Demographic and Health Survey
PSU: Primary sampling unit

## Footnotes

1 Nigeria is divided into six geopolitical zones, with each geopolitical zone comprising about six states. These are North-West, North-East, North-Central, South-West, South-East and South-South. The northern zones are generally associated with a higher poverty prevalence (especially the North-West and North-East) ([24]

2 This was not used in the decomposition analysis because the benchmark category is ambiguous as it contains both zero visit and at least four visits.
